# miR-125a Induces HER2 Expression and Sensitivity to Trastuzumab in Triple-Negative Breast Cancer Lines

**DOI:** 10.3389/fonc.2020.00191

**Published:** 2020-02-28

**Authors:** Lihi Ninio-Many, Elad Hikri, Tamar Burg-Golani, Salomon M. Stemmer, Ruth Shalgi, Irit Ben-Aharon

**Affiliations:** ^1^Department of Cell and Developmental Biology, Sackler Faculty of Medicine, Tel-Aviv University, Tel Aviv-Yafo, Israel; ^2^Davidoff Center, Rabin Medical Center, Institute of Oncology, Petah-Tiqva, Israel; ^3^Sackler School of Medicine, Tel Aviv University, Tel Aviv-Yafo, Israel; ^4^Division of Oncology, Rambam Health Care, Haifa, Israel

**Keywords:** miRNA, TNBC, ErbB2, epigenetics, migration, apoptosis

## Abstract

The EGFR/HER2 signaling network is an effective therapeutic target for HER2-positive cancers, which are known for their aggressive biological course. Evidence indicates that the EGFR/HER2 network plays a role in the aggressive basal-like subtype as well. Here, we studied the potential role of miR-125a-3p as a modulator of the EGFR/HER2 pathway in basal-like breast cancer. Over-expression of miR-125a-3p reduced the migratory capability of MDA-MB-231 cells and led to an increase in the expression of ErbB2 transcript and protein. The induced ErbB2 responded to trastuzumab and underwent internalization and subsequent intra-lysosomal degradation. Trastuzumab treatment further reduced the migratory capability and induced the apoptosis of the cells. An *in-vivo* mouse model, which supported the *in-vitro* findings, showed a synergistic effect for miR-125a-3p and trastuzumab. Trastuzumab-treated miR-125a-3p-induced tumors were significantly smaller than control induced tumors. Our findings indicate that, in the basal-like subtype of breast cancer, miR-125a-3p may act as a tumor suppressor. miR-125a-3p induces an increase in the expression of ErbB2 that may render the cells suitable for treatment with anti-HER2 therapies.

## Introduction

Gene expression profiling enables the classification of breast cancer into distinct molecular subtypes with prognostic significance based upon histological grade and the status of estrogen receptor (ER), progesterone receptor (PgR), and HER2/neu expression (HER2 or ErbB2). Based on such profiling, breast cancer is classified into the basal-like, luminal, and HER2-enriched subgroups (1). The commonly used proxy for basal-like breast cancer remains the “triple-negative breast cancer” (TNBC) phenotype, which lacks expression of the three receptors, and of which ~80 to 90% manifest the basal-like phenotype (2). This classification has therapeutic implications; luminal cancers are generally hormone receptor-positive and responsive to endocrine therapy, while the HER2-positive subtype is HER2-driven and is sensitive to targeted therapy. The basal-like subtype of breast cancer remains a clinical challenge because no effective targeted therapy has been established to date, except for PARP inhibitors, which are effective only for BRCA carriers.

ErbB2 (HER2) belongs to the human epidermal growth factor receptor (EGFR) family, which consists of four members (ErbB 1–4) and has been found in several human malignancies to be amplified at the genomic level and/or overexpressed at the protein level. Downstream ErbB signaling modules include the phosphatidylinositol 3-kinase/Akt (PKB) pathway and the Ras/Raf/MEK/ERK1/2 pathway, which involves in proliferation, survival, and growth pathways. In addition, the ErbB family induces the activation of focal adhesion kinase (FAK), which plays a critical role in cell attachment, migration, and invasion by regulating the formation and extension of cell membrane protrusions (3, 4).

MicroRNAs (miRNAs) constitute a class of small (19–25-nucleotide) non-coding RNAs that function as posttranscriptional gene regulators and are capable of exerting pronounced influence upon the translation and stability of mRNAs (5). Mounting evidence indicates that aberrantly expressed miRNAs are involved in cancer pathogenesis of solid tumors through their ability to control the expression of protein-coding tumor suppressors and oncogenes (6). In the current study, we focused on miR-125a. Though this miRNA has two mature forms derived from its 3' or 5' sequences (miR-125a-3p and miR-125a-5p, respectively), we specifically evaluated the role of miR-125a-3p.

miR-125a has been studied in several malignancies and was shown to act as a tumor suppressor. Its mode of action was linked to ErbB2 in several cancerous cells that highly express this receptor. In the breast cancer cell line SKBR3, over-expression of miR-125a down-regulates ErbB2 and ErbB3 at both the transcript and protein level, thereby reducing the cells' migration and invasion capabilities (7). This reduction was not detectable in an HER2-independent non-tumorigenic epithelial cell line (MCF10A), suggesting that miR-125a acts through a mechanism that is dependent on ErbB2 expression in the cells. In the same manner, miR-125a-5p was able to reduce the expression of ErbB2 in human gastric cancer cells that highly express ErbB2 and to suppress the proliferation of the cells. The effect was enhanced when combined with the anti-ErbB2 monoclonal antibody trastuzumab (8). To date, the functional role of miR-125a in cells expressing low levels of ErbB2 remains largely unexplored.

miR-125a-3p has been considered a tumor suppressor miRNA, indicated by its ability to regulate processes of invasion and migration in gastric and lung cancer cells (9–11). In a previous study, we showed that the miR-125a-3p level has an inverse correlation with the Gleason score of human prostate cancer tissues. It also remarkably impaired the focal adhesion sites and reduced the migratory capability of prostate cancer cells (PC3) (12).

Basal-like tumors usually exhibit an aggressive biological course with high metastatic seeding potential. Because migration and invasion pathways are likely highly pertinent in this subtype, we hypothesized that miR-125a-3p may be a potential player in carcinogenesis. In this study, we evaluated the effect of modulating MDA-MB-231 cells, a representative cell line of the triple-negative subtype, with miR-125a-3p. We show here that modulation of these cells resulted in increased expression of ErbB2 mRNA and protein. The induced ErbB2 receptor was able to undergo internalization in response to trastuzumab. In an *in-vitro* model, overexpression of miR-125a-3p hampered the migratory capability of the cells, induced apoptosis, and appeared to sensitize MDA-MB-231 cells to trastuzumab treatment, manifested by a greater extent of migration inhibition. In an *in-vivo* nude mouse model, tumors induced by injected miR-125a-3p-overexpressing cells responded to trastuzumab treatment with significant tumor shrinkage. Thus, our findings indicate that miR-125a-3p enables an initially HER2-negative cancer cell to respond to anti-HER2 therapy.

## Results

### Characterizing the Expression Profile of ER, ErbB2, and miR-125a in MDA-MB-231 Cells

**Figure 1:**
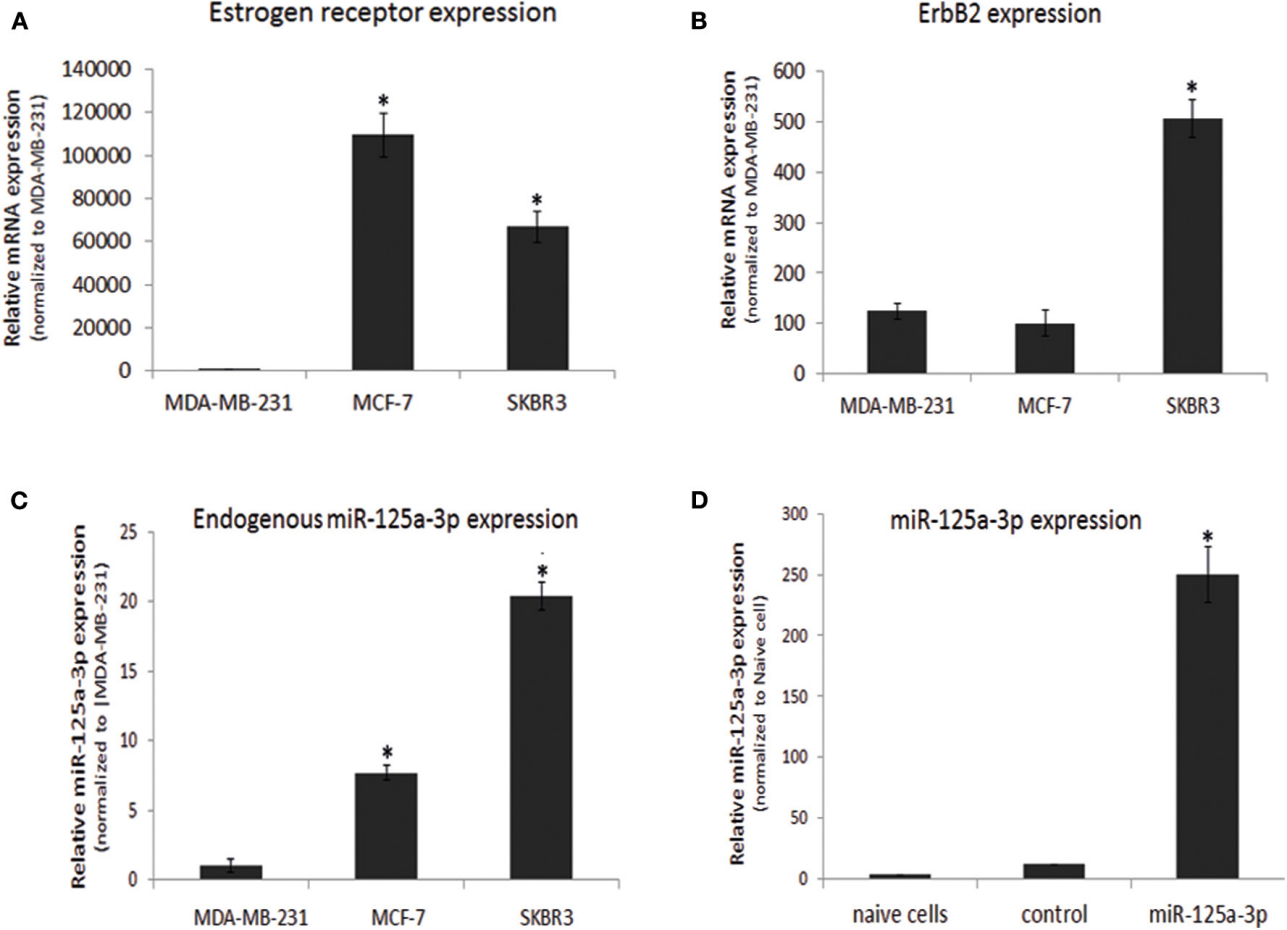
Characterization of breast cancer cell lines. **(A–C)** Three breast cancer cell lines were subjected to qPCR analysis with specific primers for **(A)** estrogen receptor, **(B)** ErbB2 calibrated with HPRT1, and **(C)** miR-125a-3p calibrated with U6 snRNA. Data were normalized to MDA-MB-231 cells. **(D)** Non-transfected MDA-MB-231 cells (naive cells) or cells transfected with either scrambled miRNA (control) or miR-125a were subjected, 48 h later, to qPCR analysis with specific primers for miR-125a-3p and for U6 snRNA as an endogenous control. All experiments were repeated three times and analyzed by a one-sample Student's *t*-test. Data are presented as mean ± SEM. *P < 0.05—significantly different from MDA-MB-231 cells **(A–C)**, or naive cells **(D)**.

In this study, we focused on the MDA-MB-231 cell line, which has the phenotype of the basal-like subtype of breast cancer. We validated the molecular characteristics of this cell line by portraying the expression profiles of ER and ErbB2 and comparing them to those of two other breast cancer cell lines: MCF-7, which corresponds to a luminal subtype, and SKBR3, which corresponds to HER2 (ErbB2)-enriched subgroups. As expected, the expression of ER (determined by qPCR) was almost undetectable in MDA-MB-231 cells and high in MCF-7 cells (Figure 1A). The expression of ErbB2 was low in MDA-MB-231 cells and high in SKBR3 cells (Figure 1B). When characterizing the expression profile of miR-125a-3p, we found that it was endogenously expressed in all cell lines, although its expression in MDA-MB-231 cells was significantly lower than in the MCF-7 and SKBR3 lines (Figure 1C). Transient transfection of MDA-MB-231 cells with miR-125a resulted in over-expression of miR-125a-3p and a non-significant increase in the expression of miR-125a-5p (data not shown) compared to control cells transfected with scrambled miRNA (control; Figure 1D).

### Overexpression of miR-125a-3p Reduces Cell Migration and Expression Level of Tumorigenic Genes

We previously showed that overexpression of miR-125a-3p impaired cell viability [HEK cells; (13)] and migration [HEK and prostate cells;(12)]. We also found that miR-125a-3p reduced the activity of Akt, FAK, Fyn, and Paxillin, key factors in the viability and migration pathways, and showed that the dynamic interplay between the actin cytoskeleton and cell adhesion sites was impaired in miR-125a-3p-overexpressing prostate cells (13).

**Figure 2:**
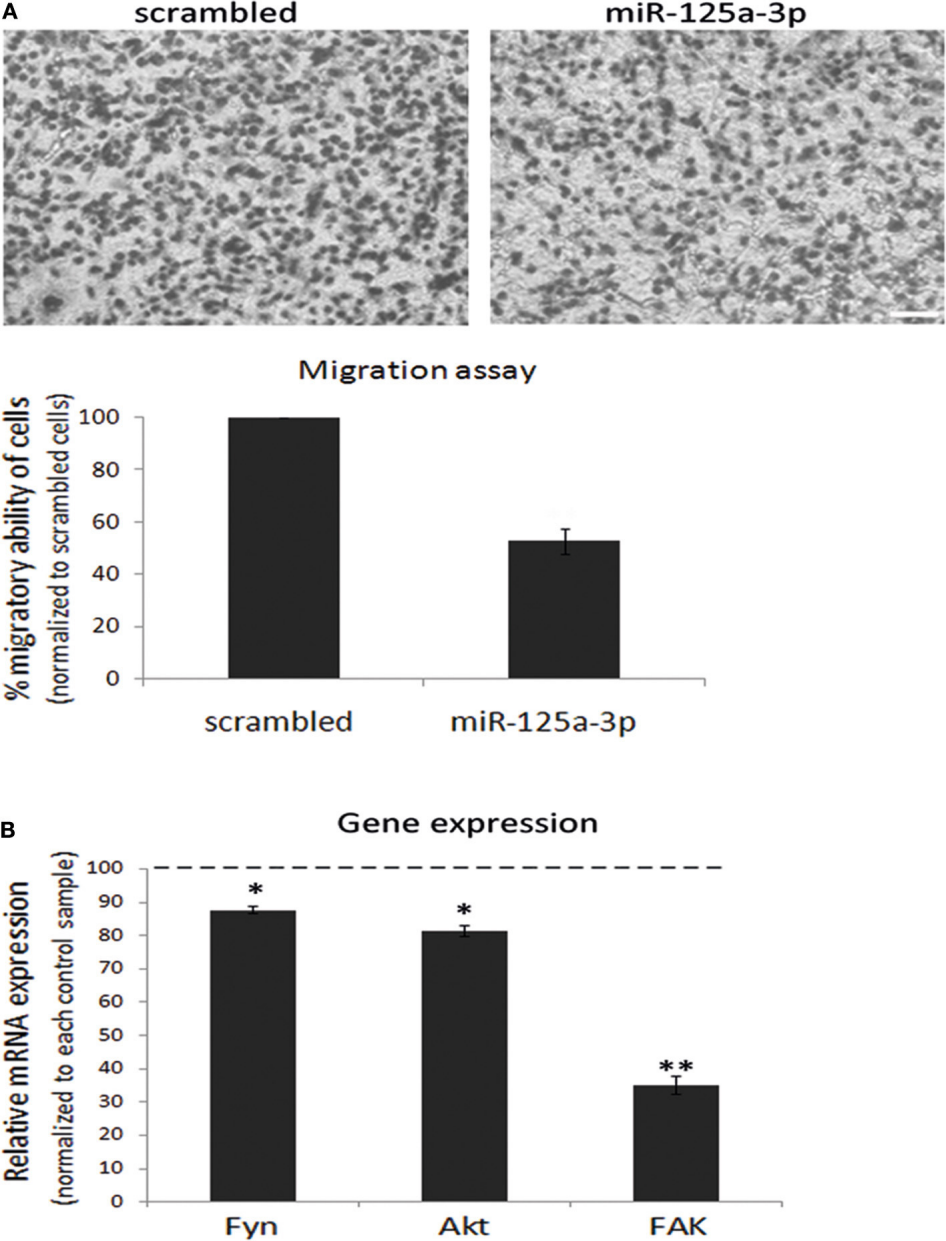
miR-125a-3p regulates the migration capability of MDA-MB-231 cells. MDA-MB-231 cells, stably expressing miR-125a-3p (miR-125a-3p) or scrambled miRNA (scrambled), were subjected to the following analyses. **(A)** Cell migration, assayed by a Transwell system for 12 h. Upper panel—representative cell culture micrographs. Bar = 50 μm. Lower panel—summary of three experiments, analyzed using Image J software. **(B)** qPCR analysis with specific primers for Fyn, FAK, and Akt; HPRT1 served as an endogenous control. The expression of each gene was normalized to its expression in control cells (100%) and is shown as mean ± SEM. The experiment was repeated three times and analyzed by a Student's *t*-test. *P < 0.05; **P < 0.01.

Since the ability of miRNAs to regulate target genes is cell type-specific, we assessed whether miR-125a-3p can regulate the proliferation and migration of MDA-MB-231 cells. To this end, we performed a Transwell assay in which we seeded an equal number of viable cells of each group and allowed the cells to migrate through the pores toward the lower chamber for 12 h. We found that miR-125a-3p caused a 40% decrease in the migration of the cells compared to cells overexpressing a scrambled (control) RNA sequence (Figure 2A) but had no significant effect on the proliferation rate of the cells (data not shown). Moreover, miR-125a-3p caused a significant decrease in the expression level of Fyn, Akt, and FAK transcripts (Figure 2B).

### Overexpression of miR-125a-3p Induces the Expression of ErbB2 in MDA-MB-231 Cells

**Figure 3:**
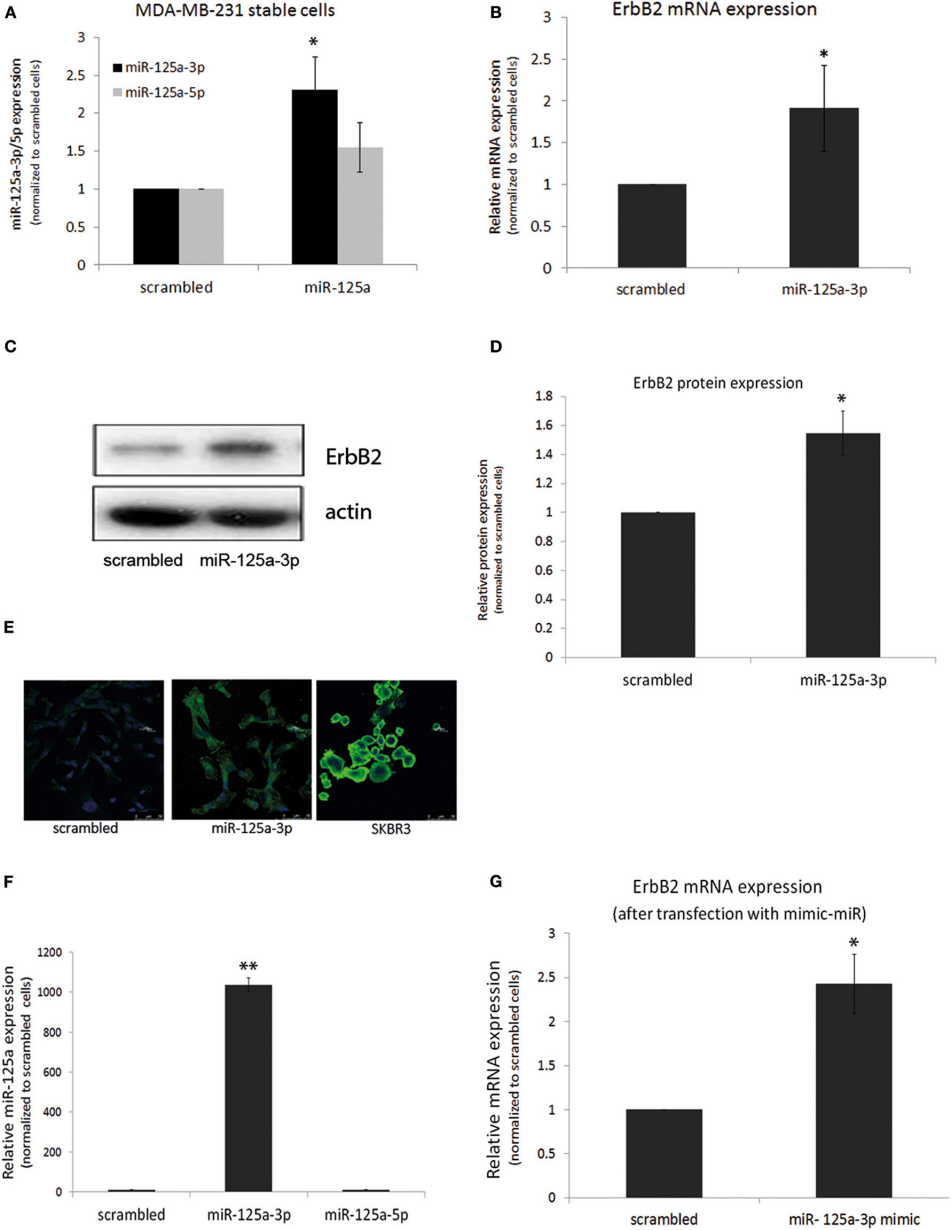
miR-125a-3p increases the expression of ErbB2 in MDA-MB-231 cells. **(A–D)** MDA-MB-231 cells, stably overexpressing either miR-125a-3p (miR-125a-3p) or scrambled miRNA as a control (scrambled), were subjected to: **(A)** qPCR with specific primers for miR-125a-3p, miR-125a-5p, and U6 snRNA as an endogenous control; **(B)** qPCR with specific primers for ErbB2 and HPRT1 as an endogenous control; **(C,D)** Western blot (WB) using anti-ErbB2 antibody and anti-actin to normalize loading; **(C)** one representative blot from analysis of three independent WB assays **(D,E)** immunofluorescence after staining with anti-ErbB2 antibody (green) and Hoechst (blue) as a nuclear marker; SKBR3 cells were used as positive control. **(F,G)** MDA-MB-231 cells, transfected with miR-125a-3p mimic (miR-125a-3p) or scrambled RNA as a control (scrambled), were cultured for 48 h and subjected to **(F)** qPCR with specific primers for miR-125a-3p, miR-125a-5p, and U6 snRNA as an endogenous control or to **(G)** qPCR with specific primers for ErbB2 and HPRT1 as an endogenous control. All experiments were repeated three times. *P < 0.05, **P < 0.01.

Because a single miRNA can simultaneously target several mRNAs belonging to a given cellular pathway (14), we looked for an upstream factor that mediates the survival and motility of oncogenic cells. We assumed that ErbB2, whose role in mediating survival, proliferation, and migration was already well-known, might be down-regulated by miR-125a-3p, as previously shown in SKBR3 cells. To further understand the effect of miR-125a-3p on the ErbB2 expression level, we assessed this effect in cells stably overexpressing miR-125a-3p. For this, MDA-MB-231 cells were infected with either miR-125a construct or with scrambled miRNA using a lentivirus system. Stable cells that overexpress scrambled miRNA are referred to as control cells (Figure 3A). Stable cells that were infected with miR-125a overexpressed miR-125a-3p, with no change in the expression level of miR-125a-5p as compared to the control; hence, they will be referred to as miR-125a-3p (Figure 3A).

Using qPCR, we found that over-expression of miR-125a-3p did not reduce but rather augmented the expression level of ErbB2 transcripts (Figure 3B) and protein (Figures 3C,D) and also the immunofluorescence staining of ErbB2 (Figure 3E).

To ensure that the induction of ErbB2 expression was mediated by the 3p isoform, cells were transfected with a miR-125a-3p mimic, a small RNA that contains only the 3p isoform sequence. qPCR validated an increase in the expression of the 3p isoform with no change in the expression of the 5p isoform (Figure 3F). The 3p-overexpressing cells also demonstrated increased expression of ErbB2 mRNA (Figure 3G).

**Figure 4:**
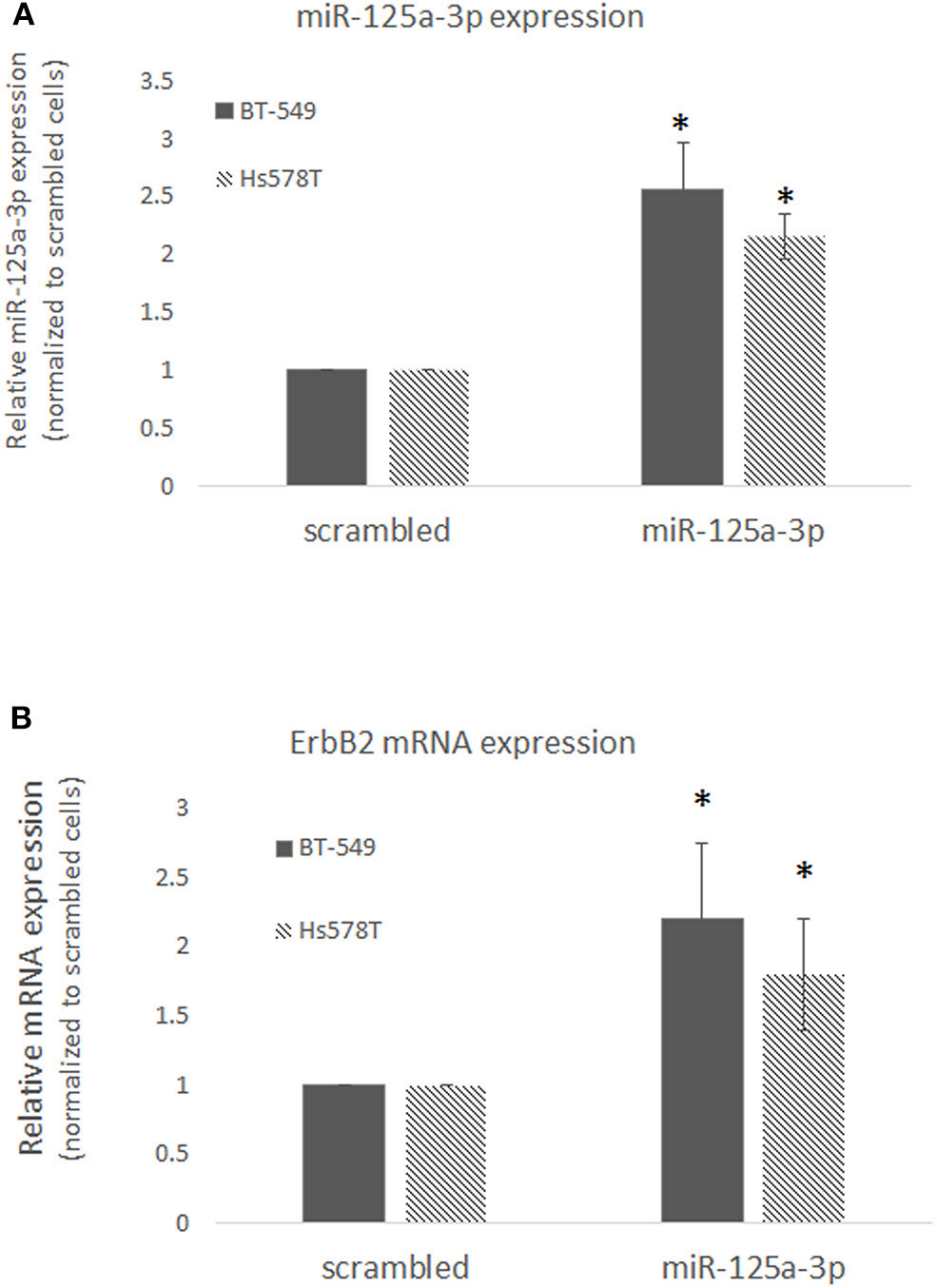
miR-125a-3p increases the expression of ErbB2 in TNBC cell lines. BT-549 or Hs578T cells transfected with either miR-125a-3p (miR-125a-3p) or scrambled miRNA as a control (scrambled) were subjected after 48 h to **(A)** qPCR with specific primers for miR-125a-3p and U6 snRNA as an endogenous control; **(B)** qPCR with specific primers for ErbB2 and HPRT1 as an endogenous control. All experiments were performed three times. *P < 0.05.

Since TNBC is highly heterogeneous, we examined the effect of miR-125a-3p on ErbB2 expression in two other TNBC cell lines, BT-549 and Hs578T, and confirmed that over-expression of miR-125a-3p in these cells induced the expression of ErbB2 mRNA (Figures 4A,B). The results indicate that isoform 3p of miR-125a contributes to the elevation of ErbB2 expression in TNBC cells.

### Trastuzumab Induces Apoptosis and Reduces Migration of MDA-MB-231 Cells Overexpressing miR-125a-3p

**Figure 5:**
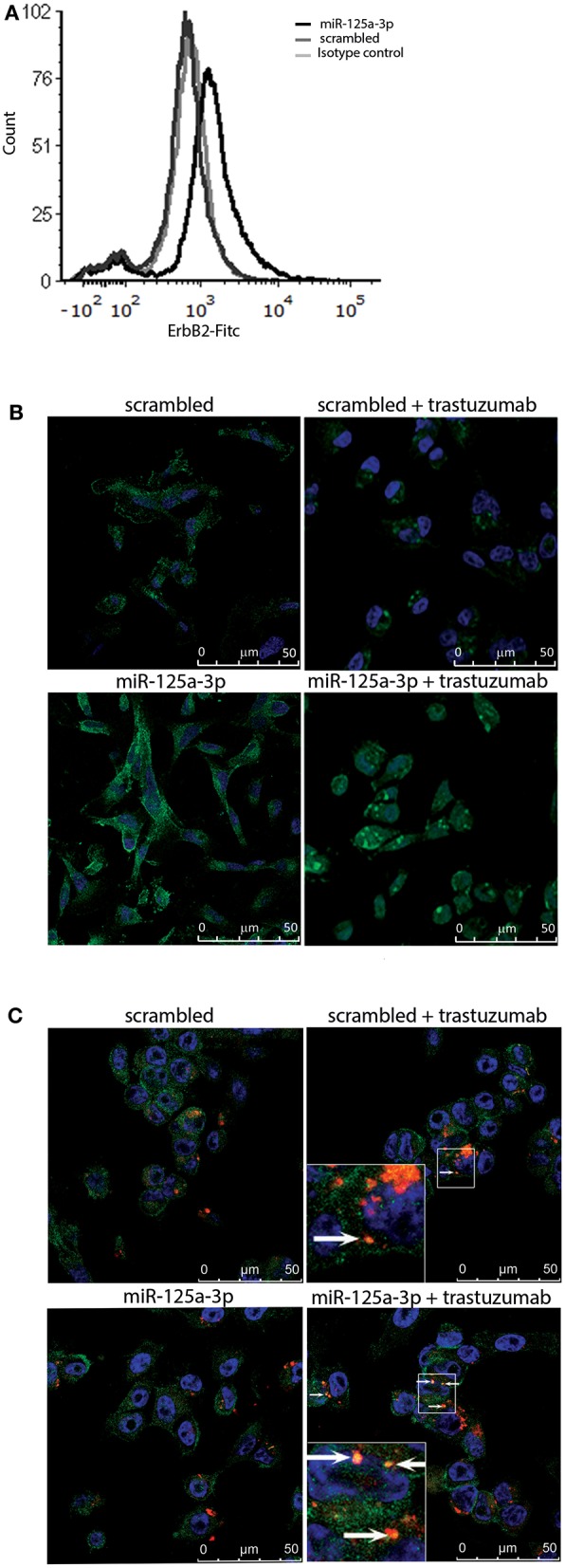
Induced-ErbB2 responds to Trastuzumab. **(A)** MDA-MB-231 cells stably expressing miR-125a-3p (miR-125a-3p) or scrambled RNA (scrambled) were stained with FITC anti-human ErbB2 IgG1 (#324404, BioLegend) or FITC mouse IgG1 isotype control (#400109, BioLegend) and analyzed by FACS, confirming their membrane expression of ErbB2. **(B,C)** Cells were treated with 100 μg/ml trastuzumab for 6 h, washed and subjected to immunofluorescence assay to track **(B)** ErbB2 internalization, immediately after incubation with trastuzumab, and **(C)** lysosomal localization 6 h after trastuzumab addition. Cells were stained with anti-ErbB2 antibody (green; **B,C**), Hoechst (blue) as a nuclear marker, and a lysosome marker (Lysotracker; red; **C**). Arrows indicate co-localization of ErbB2 and lysotracker (yellow dots). **(B,C)** Bars = 50 μm. Similar results were obtained in three independent experiments.

Based on the finding that miR-125a-3p increases the expression of ErbB2 protein, we hypothesized that overexpression of miR-125a-3p might sensitize MDA-MB-231 cells to treatment with trastuzumab, a targeted anti-HER2 therapy. To determine whether the induced ErbB2 receptors were functional and able to respond to trastuzumab, we first evaluated the expression level of membrane ErbB2. Examination by FACS following staining with anti-ErbB2 demonstrated an elevation in the expression of membrane ErbB2 in miR-125a-3p-overexpressing cells compared to control cells (Figure 5A). Next, we treated the cells with 100 μg/ml of trastuzumab for 6 h followed by immunofluorescence assay, tracking ErbB2 internalization and its localization to the lysosome. We determined that, unlike the ErbB2 receptors in control cells, most of the ErbB2 receptors at the cell surface of miR-125a-3p-overexpressing cells were internalized (Figure 5B) and some of them were co-localized, 6 h later, with a lysosomal marker (lysotracker; Figure 5C), indicating that trastuzumab induced internalization of ErbB2 and its subsequent proteolysis in the lysosome. This finding suggests that cell surface ErbB2 is elevated in miR-125a-3p-overexpressing cells and responds to trastuzumab.

**Figure 6:**
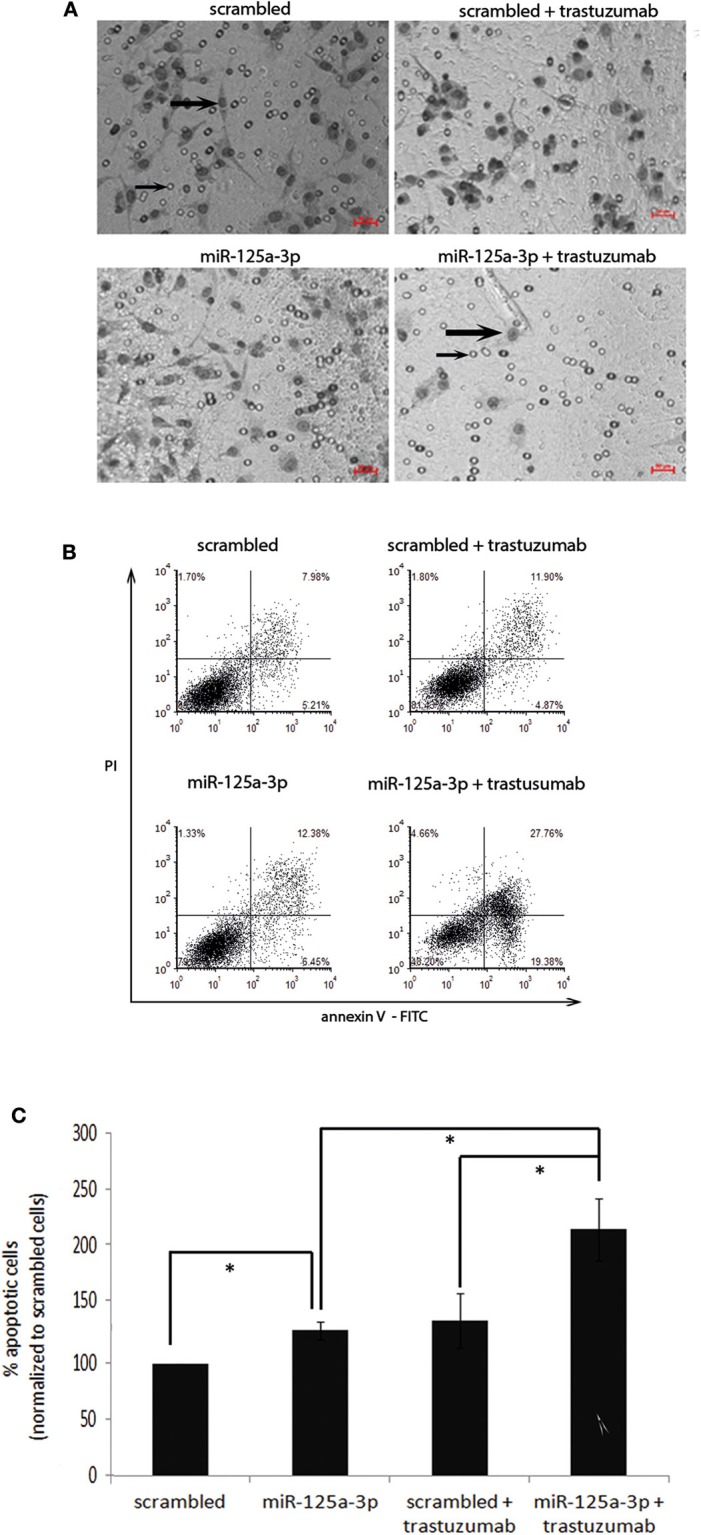
miR-125a-3p and trastuzumab have a synergistic effect in inducing apoptosis and reducing the migration reduction of MDA-MB-231 cells. **(A)** MDA-MB-231 cells, stably expressing miR-125a-3p (miR-125a-3p) or scrambled RNA as a control (scrambled), were left untreated or treated with 100 μg/ml trastuzumab for 6 h and subjected to Transwell assay. Thick arrows indicate migrating cells. Thin arrows point at the membrane pores. Bars = 50 μm. The results were analyzed using Image J software. **(B,C)** MDA-MB-231 cells, stably expressing miR-125a-3p (miR-125a-3p) or scrambled RNA (scrambled), were left untreated or treated with 100 μg/ml trastuzumab for 3 weeks and subjected to FACS analysis of apoptosis using PI and annexin staining. **(B)** Plots showing a representative assay. **(C)** Graph summarizing three FACS analyses. Bars are mean ± SEM analyzed by one-sample *t*-test. *P < 0.05—significantly different from control value. **(A,B)** Similar results were obtained in three independent experiments.

After validating the functionality of the receptor, we next examined the effect of trastuzumab on the migratory capability of the cells. We therefore treated stable miR-125a-3p or cells overexpressing the scrambled construct with 100 μg/ml of trastuzumab for 6 h, seeded an equal number of viable cells from each group, and tested their migration by Transwell. We found a 21% decrease relative to untreated miR-125a-3p cells in the number of migrating trastuzumab-treated miR-125a-3p-overexpressing cells and no significant change in the number of migrating cells between trastuzumab-treated and untreated control cells (Figure 6A). These results indicate that miR-125a-3p and trastuzumab have a synergistic effect of inhibiting cell migration.

In order to examine whether miR-125a-3p and trastuzumab also have a long term-synergistic effect on the viability of cells, we cultured stable cells overexpressing miR-125a-3p or scrambled miRNA alone or in the presence of 100 μg/ml trastuzumab for 21 days and analyzed their apoptosis by FACS assay. We found that miR-125a-3p alone was effective in increasing the apoptosis rate of the cells (by ~30% compared to that of control cells). Trastuzumab treatment of the miR-125a-3p-overexpressing cells caused a significant increase in the rate of apoptosis, an increase of 60% compared to untreated miR-125a-3p-overexpressing cells or to control cells treated with trastuzumab. There was no significant difference in the apoptosis rate between cells treated with trastuzumab alone and untreated control cells (Figures 6B,C).

### miR-125a-3p and Trastuzumab Have a Synergistic Effect in Reducing Tumor Growth

**Figure 7:**
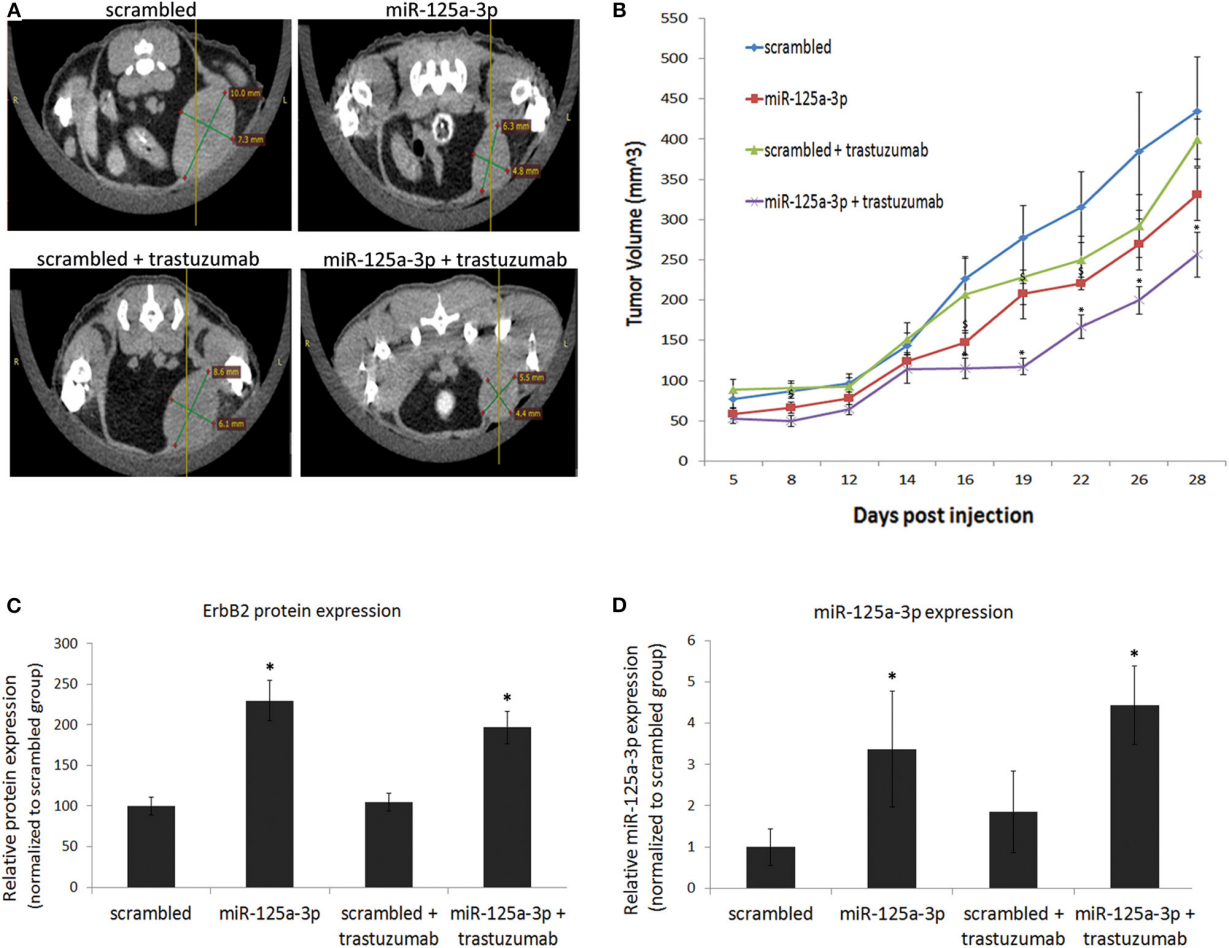
miR-125a-3p and trastuzumab have a synergistic effect on tumor growth. Cells (1.5 × 10^6^), stably expressing miR-125a-3p (miR-125a-3p) or scrambled miRNA as a control (scrambled), were injected into the mammary fat pads of 6 week-old mice. Starting after 1 week, the mice were treated twice a week for 28 days with 10 mg/kg trastuzumab or with vehicle (control). Each group contained six mice. **(A)** Representative CT images taken after 16 days of trastuzumab treatment. **(B)** Graphical representation of tumor growth, shown as mean ± SEM. Mice were sacrificed on the 28th day, their tumors were excised, and the expression of ErbB2 and miR-125a-3p was analyzed by WB and by qPCR (**C,D**, respectively). Data were analyzed by a Student's *t*-test. *P < 0.05—significantly different from “scrambled.” *P < 0.05—significantly different from miR-125a-3p.

To examine whether the synergistic effect of miR-125a-3p and trastuzumab also occurs in an *in-vivo* model, we established a mouse model of breast cancer. MDA-MB-231 cells, stably expressing miR-125a-3p or scrambled miRNA (as control), were injected into the mammary fat pads of nude mice. Starting 1 week post-injection, mice were treated twice a week, for the next 28 days, with 10 mg/Kg trastuzumab or saline as a control, and tumor size was measured by Computed Tomography (CT; Figure 7A) or caliper (Figure 7B). Treatment with trastuzumab had no significant effect on tumors originating from control cells but had a synergistic effect on the miR-125a-3p-induced tumors, which were significantly smaller than untreated miR-125a-3p-induced tumors (Figures 7A,B). At the conclusion of the experiment, we used qPCR to examine the expression level of miR-125a-3p and ErbB2 in the extracted tumors and showed that overexpression of ErbB2 and miR-125a-3p in miR-125a-3p-induced tumors was preserved (Figures 7C,D, respectively). Taken together, our findings suggest that the combination of miR-125a-3p and trastuzumab could be a novel therapeutic approach against triple-negative breast cancer.

## Discussion

The EGFR/HER2-derived pathway has been implicated in a variety of cellular processes, including cell motility, invasiveness, angiogenesis, resistance to apoptosis, and metastatic potential (15). This pathway plays a key role in the mechanism of targeted therapies for HER2-overexpressing cancers and serves as a prognostic and predictive biomarker in breast and gastric cancer. Nevertheless, there is evidence that the EGFR/HER2 network is also essential in basal-like subtypes (16). The basal-like subtype represents a subset of cancer with a particularly aggressive phenotype and poor clinical response to biological therapy (17).

Our observation that overexpression of miR-125a-3p in a cell line of the basal-like breast cancer subtype resulted in high expression of ErbB2 prompted us to further explore whether modulation with miR-125a-3p may sensitize these cells to the highly effective anti-HER2 therapies. We determined that the newly expressed ErbB2 was functional and responsive to trastuzumab treatment, showing that after treatment with trastuzumab, a portion of the receptors underwent endocytosis and lysosomal internalization. We demonstrated, in an *in-vitro* model, that a combination of trastuzumab and high expression of miR-125a-3p had a synergistic effect of reducing the migration of the cells and inducing apoptosis. Trastuzumab treatment resulted in a 120% increase in the rate of apoptosis in miR-125a-3p-overexpressing cells, whereas there was only a 50% increase in trastuzumab-treated control cells. Accordingly, we found using an *in-vivo* model that the tumor burden of mice bearing miR-125a-3p-induced-tumors treated with trastuzumab was significantly decreased compared to that of trastuzumab-treated mice bearing tumors induced by control cells. In all, our findings suggest that miR-125a-3p, alone or in combination with trastuzumab, could potentially serve as a novel therapeutic approach for basal-like breast cancer.

The role of miR-125a as a regulator of ErbB2 in both breast and gastric cancer cells has already been established. Scott et al. showed that miR-125a-5p binds to and down-regulates the expression of ErbB2 and ErbB3 in SKBR3 cells (7). A similar interaction was observed in ErbB2-highly expressing gastric cancer cells, as miR-125a-5p was shown to repress ErbB2 expression and in addition, to be inversely correlated with expression of ErbB2 in clinical samples (8). In these two papers, the manipulation of the cells was conducted with miR-125a, which could result in the induction of both the 5p and the 3p isoforms, though neither the expression of miR-125a-3p nor its effect on ErbB2 expression was examined. Hence, the effect of miR-125a-3p on ErbB2 expression remained to be elucidated.

The mechanism by which miR-125a-3p induces ErbB2 expression is not clear yet. We can suggest two possible mechanisms. The first one derives from the delicate reciprocal balance between miR-125a-3p and miR-125a-5p. It has been shown that these two isoforms have opposing effects on the invasiveness and migratory ability of lung cancer cells (18) but work similarly to inhibit the proliferation of gastric cancer cells (11). In general, one of the strands of miR-125a is incorporated into the RNA-induced silencing complex (RISC), thereby repressing gene expression, while the passenger strand is degraded. Hence, it is possible that diverting the balance between the two isoforms toward formation of the 3p isoform causes decreased formation of the 5p isoform and, consequently, induces expression of ErbB2. In our experimental system, we were unable to demonstrate a consistent relationship between the two isoforms over various cell lines. Recent reports suggest that incorporation of either strand (5p or 3p) into the RISC complex is possible (19), thereby adding another layer of complexity. Further study is required in order to understand how the balance between the 3p and 5p isoforms affects ErbB2 expression.

Another putative mechanism may arise from direct regulation of ErbB2 expression by miR-125a-3p. miRNAs can induce gene expression by binding the target gene sequence at a site that is not merely located within its 3'UTR. For example, miR-373 was shown to induce the expression of E-cadherin by specific binding to a target site located within its promoter (11). In our study, in searches for complementation between miRNA-seed sequences and the 3'UTR of target genes, ErbB2 was not predicted to be a miR-125a-3p-target gene. Whether miR-125a-3p induces the expression of ErbB2 through complementation over the entire ErbB2 gene sequence remains an open question.

After characterizing the phenotype and biological behavior of miR-125a-3p- overexpressing cells, we observed that the induced expression of ErbB2 was not translated into higher aggressiveness of the cells; in fact, the apoptosis rate was enhanced, and the migratory capability of the cells was hampered. Due to the dual effect of miR-125a-3p in inducing ErbB2 expression on the one hand and inhibiting cell migration on the other hand, we postulate that the decreased migratory capability may derive mainly from the effect exerted by miR-125a-3p on ErbB2-downstream pathways. The three well-characterized signaling pathways induced through ErbB receptors are Ras-MAPK, PI3K-Akt, and PLC-PKC. We demonstrated that overexpression of miR-125a-3p down-regulated the expression of Akt, a key regulator of survival during cellular stress (20, 21), making miR-125a-3p a unique potential target for anti-cancer therapy (22).

Other factors such as Fyn and FAK, which are commonly activated in breast cancer and participate in the induction of migration, were also down-regulated by miR-125a-3p, as we previously demonstrated in HEK cells (13) and prostate cancer cells (12). Fyn is a direct target of miR-125a-3p (13). In contrast, Akt and FAK are not predicted to be direct targets of miR-125a-3p but are Fyn-downstream proteins, and their regulation likely occurs in an indirect manner. We conclude that though miR-125a-3p induces ErbB2 expression, it suppresses the downstream signaling pathways, reducing the migratory capability of the cells and acting as a tumor suppressor miRNA in MDA-MB-231 cells.

The inhibitory effect on migration is consistent with former studies, among which is our study in prostate cancer cells (12), showing that the migration of miR-125a-3p-overexpressing prostate cancer cells was compromised by 50% and that the level of miR-125a-3p is inversely correlated with the Gleason score of tissue samples obtained from patients diagnosed with prostate cancer. This inhibitory effect was also demonstrated in glioblastoma cells, both *in-vitro* and *in-vivo* and in liver cancer, as low levels of miR-125a-3p expression were found to be associated with enhanced malignant potential, as manifested by tumor size and metastasis (11).

Clinical evidence suggests that even a minor elevation of ErbB2 expression increases the tumor response to anti-HER2 treatment due to the effect of the latter on activation of the antibody-dependent cellular immunity system and on local angiogenesis. The *in-vivo* model used here was established in nude mice that lack essential T-cell immunity; the observed effect may be further enhanced when this experimental platform is established in immune-intact mice.

## Conclusions

Our findings indicate that miR-125a-3p is capable of inducing a shift in the involvement of the ErbB2 pathway in the basal-like subtype of breast cancer, thereby sensitizing the cells to anti-HER2 therapies. Our study proposes a means to expand the patient population that may benefit from anti-HER2 therapies.

## Materials and Methods

### Cell Lines and Treatments

Adherent cultures of human MDA-MB-231, MCF-7, SKBR-3, Hs578T, BT-549, and HEK 293T cell lines were obtained from the American Type Culture Collection (Manassas, VA, USA) and tested regularly for mycoplasma. BT-549 cells were maintained in RPMI culture medium; all other cells were maintained in Dulbecco's modified Eagle's medium (DMEM; Biological Industries, Beit-Ha'emek, Israel). Both media were supplemented with 10% fetal calf serum (FCS), 2 mM L-glutamine, and antibiotics (Biological Industries). All the cells were cultured at 37°C in a humidified atmosphere of 5% CO_2_ in air and maintained for <20 passages. As indicated, cells were treated with 100 μg/ml trastuzumab, also known as Herceptin (Genentech Inc., San Diego, CA, USA).

### Manipulation of Gene Expression

#### Plasmid Transfection

Cells were transfected with plasmids using Lipofectamine 2000 Transfection Reagent (Invitrogen, Waltham, MA, USA) according to the manufacturer's instructions. For transfection of the miR-125a-3p mimic, 20 nM of mature miR-125a-3p and 5 μl of Lipofectamin 2000 Transfection Reagent were used. Complete medium was added 24 h after transfection for an additional 24 h of culture before subjecting the cells to subsequent analyses.

#### Infection

The miR-125a-3p sequence was sub-cloned from miR-Vec plasmid into CD-515b-1 plasmid (pCDH-CMV-EF1-Higro vector; Tarom Applied Technologies, Israel). HEK 293T cells were co-transfected with pCDH-CMV-miR-125a-3p-EF1-Higro vector and with the compatible packaging plasmids (pPACKH1 lentivector packaging kit, #LV-500A-1; SBI system biosciences, CA, USA). Cells were incubated for 48 h after transfection and then centrifuged, and the supernatant that contained lentiviral particles was collected. MDA-MB-231 human breast cancer cells were infected with the lentiviral particles by incubation with the culture media. miR-125a-3p-positive cells were selected 7 days later by their resistance to Hygromycin (AG Scientific., San Diego, CA, USA), and their overexpression of miR-125a-3p was validated by qPCR.

### Evaluation of RNA

#### RNA Isolation and Reverse Transcription (RT)

Total RNA was extracted from the cells by Trizol (Invitrogen) according to the manufacturer's instructions. Reverse transcription (RT) for analysis of gene expression or miRNA expression was carried out by high capacity cDNA RT kit (Applied Biosystems, Carlsbad, CA, USA; 10-ng RNA fractions).

#### Quantitative Reverse Transcriptase Polymerase Chain Reaction (q-RT-PCR)

All RT reactions were carried out using the StepOnePlus Real-Time PCR System (Applied Biosystems).

#### qPCR for Gene Expression

The reactions were conducted using SYBR Green dye (Applied Biosystems) according to the manufacturer's protocol. The following primers were used for the analyses:

ErbB2 (forward primer: 5'-GGTCCTGGAAGCCACAAGG-3', reverse primer: 5'-GGTTTTCCCACCACATCCTCT-3')

ER (forward primer: 5'-TGATGAAAGGTGGGATACGA-3', reverse primer: 5'-AGCTCTCATGTCTCCAGCAG-3')

Fyn (forward primer: 5'-GGACATGGCAGCACAGGTG-3', reverse primer: 5'-TTTGCTGATCGCAGATCTCTATG-3')

Akt (forward primer: 5'- ACGTGGCTATTGTGAAGGAG-3', reverse primer: 5'- CATTCTTGAGGAGGAAGTAGCG-3')

FAK (forward primer: 5'- AAATACGGCGATCATACTGGG-3', reverse primer: 5'- TTGGCCTTGACAGAATCCAG-3')

Hypoxanthine phosphoribosyltransferase 1 (HPRT1) as endogenous control (forward primer: 5'-TGACACTGGCAAAACAATGCA-3', reverse primer: 5'-GGTCCTTTTCACCAGCAAGCT-3').

#### qPCR for miRNA Expression

miR-125a-3p (Assay ID: 2199), miR-125a-5p (Assay ID: 2198), and U6-snRNA (Assay ID: 001973) were measured by the TaqMan miRNA kit (Applied Biosystems) according to the manufacturer's instructions. Mature miRNAs were normalized to U6-snRNA. Relative expression was calculated using comparative ΔCt.

#### Western Blot

Samples were subjected to sodium dodecyl sulfate polyacrylamide gel electrophoresis (SDS-PAGE) as previously described (13) and immunoblotted with the appropriate primary antibodies: anti-ErbB2 (#29D8; Cell Signaling Technology, MA, USA) or anti-actin (#MAB1501; Millipore, Temecula, CA, USA). Blots were visualized using ECL (Pierce Pico ECL kit).

#### Flow Cytometry

Cells were suspended in FACS buffer (5% FCS, 0.05% sodium azide in PBS) and incubated with FITC anti-human ErbB2 IgG1 (#324404, BioLegend) or FITC mouse IgG1 isotype control (#400109, BioLegend) antibodies for 30 min at 4°C in the dark and then washed prior to analysis. Samples were collected with FACScanto (BD PharMingen) and analyzed with FCS Express software (De Novo Software); 50,000 total events were recorded per sample.

#### Immunofluorescence Staining

Immunofluorescence was performed as described previously (12). Briefly, cells were stained with FITC-conjugated anti-ErbB2 (#ab31891; Abcam, Cambridge, UK), washed, stained with Lysotracker Deep Red (#L12492; Thermo Fisher Scientific, Waltham, MA, USA) and with Hoechst 33342 (1 mg/ml; Sigma-Aldrich, Rehovot, Israel), and analyzed on an Zeiss LSM 510 laser confocal scanning microscope (Carl Zeiss, Oberkochen, Germany) or a Leica TCS STED (Stimulated Emission Depletion) microscope (Leica, Wetzlar, Germany).

#### Migration Assays

Migration assays were performed as previously described (13). Briefly, samples of MDA-MB-231-overexpressing miR-125a-3p or scrambled RNA, treated/untreated with trastuzumab, were stained with Trypan blue, and their viability was examined using a Countess automated cell counter (Invitrogen). For migration assay, live cells (2^*^105) were pre-incubated in FCS-free DMEM in the upper wells of a Transwell plate (24 wells, 8 μm pore size membranes, Corning 3422; Corning, NY, USA). After 6 h, 350 μl of DMEM with 20% FCS was added to the lower well, and cells were allowed to migrate during a 12 h incubation period at 37°C in 5% CO_2_ in air. The migrating cells at the bottom of the membrane were visualized by fluorescence microscopy, photographed, and counted using Image J software.

#### Annexin PI Staining for Apoptosis Analysis

Cells were trypsinized, washed 3 times in cold PBS, re-suspended for 15 min at room temperature in 100 μl buffer (MEBCYTO Apoptosis Kit, #4700; MBL International, MA, USA) containing 10 μl Annexin V-FITC and 5 μl PI. The cells were subjected to FACS analysis, and the readings were analyzed using FCS software.

#### *In vivo* Assays

MDA-MB-231 cells (1.5 × 10^6^), stably expressing miR-125a or scrambled miRNA, were mixed with an equal volume of Matrigel (BD Matrigel, BD Biosciences, CA, USA) and injected into the mammary fat pad of 6 week-old female athymic nude mice. Tumor size was measured twice a week with a caliper, in addition to a weekly CT scan by TomoScope®. Tumor volume was calculated using the formula: 1/2^*^[length (mm)] × [width (mm)]^2^. When tumors reached a volume of 100 mm^3^, the mice were randomly allocated to experimental and control groups and were injected intraperitoneally (i.p.) twice a week with 10 mg/kg trastuzumab or vehicle (control) for 28 additional days.

All *in vivo* procedures were performed in compliance with the guidelines of the Sackler School of Medicine, Tel Aviv University; protocols were approved by the Institutional Animal Care and Use Committee (IACUC).

### Data Analysis and Statistics

Data are expressed as mean ± SEM. Individual comparisons were performed using the Student's *t*-test. *P*-value of 0.05 was considered statistically significant.

## Data Availability Statement

All datasets generated for this study are included in the article/supplementary material.

## Ethics Statement

The animal study was reviewed and approved by the Institutional Animal Care and Use Committee (IACUC) of Tel Aviv University.

## Author Contributions

LN-M and IB-A developed the concept and wrote the manuscript. LN-M designed the experiments. LN-M, EH, and TB-G carried out the experiments, data organization, and statistical analyses. SS participated in analyzing and discussing the results. RS conceived the study, participated in its design and coordination, helped draft the manuscript, and supervised the study. All authors read and approved the final manuscript.

### Conflict of Interest

The authors declare that the research was conducted in the absence of any commercial or financial relationships that could be construed as a potential conflict of interest.

## Abbreviations

TNBC: triple-negative breast cancer
EGFR: epidermal growth factor receptor
FAK: focal adhesion kinase
miRNA: microRNA
miR-125a-3p: miRNA-125a-3p
qRT-PCR: quantitative reverse transcriptase polymerase chain reaction
WB: Western blot.
